# Follow-up of men with a PI-RADS 4/5 lesion after negative MRI/Ultrasound fusion biopsy

**DOI:** 10.1038/s41598-022-17260-6

**Published:** 2022-08-10

**Authors:** Kira Kornienko, Miriam Reuter, Andreas Maxeiner, Karsten Günzel, Beatrice Kittner, Maximilian Reimann, Sebastian L. Hofbauer, Laura E. Wiemer, Robin Heckmann, Patrick Asbach, Johann Jakob Wendler, Martin Schostak, Thorsten Schlomm, Frank Friedersdorff, Hannes Cash

**Affiliations:** 1 Department of Urology, Charité-Universitätsmedizin Berlin, Hardenbergstr.8, 10623 Berlin, Germany; 2 Department of Urology, Vivantes Am Urban, Berlin, Germany; 3 Clinic for Radiology, Charité-Universitätsmedizin Berlin, Berlin, Germany; 4 Department of Urology, University Magdeburg, Magdeburg, Germany; 5 PROURO, Berlin, Germany

**Keywords:** Medical research, Outcomes research, Prostate, Urology, Prostate cancer

## Abstract

Magnetic resonance imaging/Ultrasound **(**MRI/US) fusion targeted biopsy (TB) in combination with a systematic biopsy (SB) improves cancer detection but limited data is available how to manage patients with a Prostate Imaging-Reporting and Data System (PI-RADS) ≥ 4 lesion and a negative biopsy. We evaluate the real-world management and the rate of clinically significant Prostate Cancer (csPCa) during follow-up. 1546 patients with a multi-parametric MRI (mpMRI) and a PI-RADS ≥ 3 who underwent SB and TB between January 2012 and May 2017 were retrospectively analyzed. 222 men with a PI-RADS ≥ 4 and a negative biopsy were included until 2019. For 177/222 (80%) complete follow-up data was obtained. 66/84 (78%) had an initial PI-RADS 4 and 18 (22%) a PI-RADS 5 lesion. 48% (84/177) received a repeat mpMRI; in the follow-up mpMRI, 39/84 (46%) lesions were downgraded to PI-RADS 2 and 11 (13%) to PI-RADS 3; three cases were upgraded and 28 lesions remained consistent. 18% (32/177) men underwent repeated TB and csPCa was detected in 44% (14/32). Our study presents real world data on the management of men with a negative TB biopsy. Men with a positive mpMRI and lesions with high suspicion (PI-RADS4/5) and a negative targeted biopsy should be critically reviewed and considered for repeat biopsy or strict surveillance. The optimal clinical risk assessment remains to be further evaluated.

## Introduction

The multi-parametric magnetic resonance imaging (mpMRI) diagnostic pathway followed by a MRI/Ultrasound (MRI/US) fusion guided targeted biospsy (TB) is becoming the new standard for men at risk of prostate cancer (PCa). The strong data from studies focusing on a primary mpMRI have led to the updated recommendation by EAU and AUA guidelines towards an indication of a mpMRI for both the primary and repeat biopsy setting^1–3^.

The combination of a TB with a SB leads to improved cancer detection. Nevertheless, about 30% of TB remain negative and the management of this subgroup currently is unclear^4–7^. Guidelines recommendations on this subgroup of men are currently lacking and decisions are often made individually^1^.

A majority of systematic biopsies in biopsy- naïve men remain negative and clinically significant (cs) PCa might be overseen^8–10^. A pre-biopsy mpMRI warrants filtering biopsy-naïve men under suspicion of PCa as a first triage test but if suspicion persists despite a negative MRI adding PSA density (PSAD) as an additional factor almost excludes the detection of csPCa if it is less than 0.15 ng/ml^11–13^.

A recent Cochrane analysis by Drost et al. further confirmed the superiority of the mpMRI pathway^14^. The mpMRI is more sensitive for clinically significant cancer and can avoid a biopsy in 27% of men, therefore reducing the rate of overdiagnosis of clinically insignificant PCa by 31%^11^. Nonetheless, a variability of mpMRI quality and published detection rates for each Prostate Imaging-Reporting and Data System (PI-RADS) score persists, hence leading to a range of PCa detection rates^15^. Despite the improved mpMRI pathway and a suspicious lesion on mpMRI, approximately 33–52% of men will have negative histopathology^12,16^.

This retrospective study evaluated the management of men with PI-RADS 4 and 5 lesions after a negative MRI/US TB in a real-world setting of a consecutive cohort.

## Patients and methods

### Study population

Out of a prospective database of 1546 men who underwent transrectal MRI/US TB and SB due to suspicion of prostate cancer at Charité-Universitätsmedizin Berlin between January 2012 and May 2017, we retrospectively analyzed men with a negative biopsy of PI-RADS Score ≥ 4 lesions. Follow-up data were extracted from patient files where available and by a patient questionnaire. The questions included inter alia post-biopsy patient guidance and the indication for a repeat mpMRI or biopsy. The indication was made by the treating outpatient urologist as it is the standard of care in Germany.

Patient meta-data was collected in accordance with the standards of reporting for MRI-TB studies (START) checklist in a prospective database^17^. Subgroups of this cohort were included in previous analyses^17^. All patients signed a written informed consent for the intervention, data acquisition, and data appraisal. The study was performed according to the Declaration of Helsinki and authorized by the Institutional Review Board of the Charité-Universitätsmedizin Berlin.

### Multiparametric imaging control

All patients received a 3-T mpMRI (Magnetom Skyra; Siemens Medical Systems, Erlangen, Germany) at Charité-Universitätsmedizin Berlin. The MRI protocol always comprised multi- planar (axial and coronal) high spatial resolution T2-weighted turbo spin-echo sequences (T2w TSE), axial T1-weighted images, axial diffusion-weighted images (DWIs; measured b-values 0.400 and 800 s/mm^2^, calculated b-value of 1 400 s/mm^2^) and gadolinium-based dynamic contrast-enhanced (DCE) sequences. T2w imaging and DWIs were performed in all patients and DCE MRI in most patients. In compliance with the guidelines of the European Society of Urogenital Radiology (ESUR) the evaluation and validation of the mpMRI image data were performed or supervised by a team of experienced expert radiologists at Charité-Universitätsmedizin Berlin using PI-RADS version 2 (v2). Lesions initially rated using PI-RADS V1 were re-rated using V2 for the analysis. Experienced radiologists were defined by the consensus statement with a minimum number read of 1000 mpMRIs and a yearly read over 200^18^. Considering clinical routine, radiologists were not blinded to clinical data. For patients with multiple lesions, the maximal PI-RADS score was used for further analysis.

### MRI/US fusion-guided TB and SB

In accordance with the EAU guidelines at the time of the biopsy, the transrectal interventions were performed under antibiotic prophylaxis with a fluoroquinolone (ciprofloxacin) by a team of experienced urologists. MRI/US fusion-guided TB of the prostate was performed first, using the high-end US machine HiVison Preirus (Hitachi Medical Systems, Tokyo, Japan) or Aplio 500 (Toshiba, Otawara, Japan) with endocavity endfire probes (11C3, Toshiba; EUP V53 W; Hitachi Medical Systems) or a biplane probe transrectal (EUP CC531, Hitachi Medical Systems), as described previously^6^. Per PI-RADS lesion 3 targeted biopsies were obtained. Subsequently, transrectal SB was performed with 10 cores from left/right apex, left/right lateral midgland, left/right base, left/right ventral and left/right paraurethral.

All cores were potted and documented separately and were examined and analyzed by a certain team of experienced pathologists at Charité-Universitätsmedizin Berlin.

### Definition of clinically significant prostate cancer

Clinically significant prostate cancer was defined as Gleason score ≥ 3 + 4 = 7 (International Society of Urological Pathology [ISUP] grade 2). Gleason scoring was based on the highest grade detected on histological analysis.

### Statistical analyses

Continuous variables were described using medians and interquartile ranges (IQR), whereas categorical variables were characterized using proportions. All descriptive analyses were performed using the Statistical Package for the Social Sciences (SPSS) software version 25 for Mac OS (SPSS Inc., IBM Company, Chicago, IL, USA).

### Research involving human participants

Approval was obtained from the ethics committee of Charité University Medicine Berlin. The procedures used in this study adhere to the tenets of the Declaration of Helsinki.

### Informed consent

Informed consent was obtained from all individual participants included in the study.

## Results

**Figure 1:**
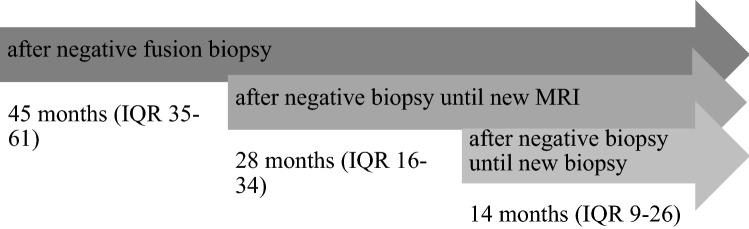
Timeline of Follow-up.

**Figure 2:**
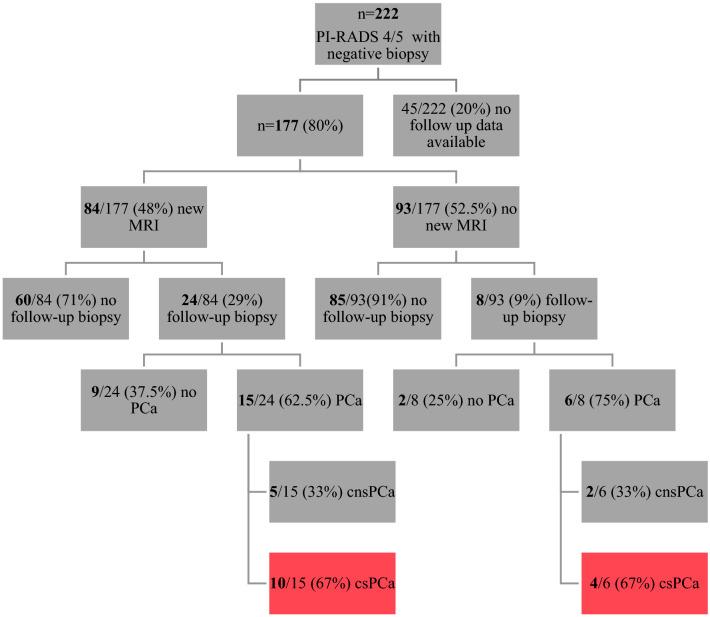
Follow-up specification in a real-life setting.

The definitive cohort included a total number of 222 men with a PI-RADS score of 4 or 5 and a negative TB in combination with a SB. Complete Follow-up data was available for 177/222 (80%) men with a median follow-up of 45 months (IQR 35–61) (Fig. 1 and 2).

The median patient age was 66 years (IQR 59–70), PSA level was 8 ng/ml (IQR 6–13), PSAD was 0.13 ng/ml^2^ (IQR 0.09–0.21) and the median prostate volume was 63 ml (IQR 47–86). 81/222 (36%) of men had one prior negative biopsy, 52 (23%) had two and 39 (18%) three or more previous negative biopsies (Table 1).

**Table 1:** Patient demographics, clinical characteristics and mpMRI findings (n = 222).

	**Value**	**IQR/%/SD**
Median (IQR) age, years	66	59–70
Median (IQR) prostate volume, ml	63	47–86
Median (IQR) pre-biopsy PSA, ng/ml	8	5.94–12.73
Median (IQR) PSAD, ng/ml^2^	0.13	0.09–0.21
**Number of previous biopsies n/222 (%)**		
No prior biopsy	50	23
1 prior biopsy negative for cancer	81	36
2 prior biopsies negative for cancer	52	23
≥ 3 prior biopsies negative for cancer	39	18
Median (IQR) number of lesions per patient	1	1–2
Median (IQR) lesion size (maximum diameter), mm	12	9–20

The distribution of the lesion as prior to the initial TB at was PI-RADS 4 in 182/222 (82%) and PI-RADS 5 in 40/222 (18%) patients and 145/177 (82%) PI-RADS 4 lesions and 32/177 (18%) PI-RADS 5 lesion for men with follow-up. A repeat mpMRI (after the initial negative TB) was performed in 84/177 (47%) men within a median time of 28 months (IQR 16–34) and led to a repeat TB in 24/177 (14%) cases. On follow-up 93/177 (53%) patients had no new mpMRI; 8 (5%) received a follow-up TB nonetheless due to a rising PSA and/ or a suspicion in the digital rectal examination (DRE) (Fig. 2).

**Figure 3:**
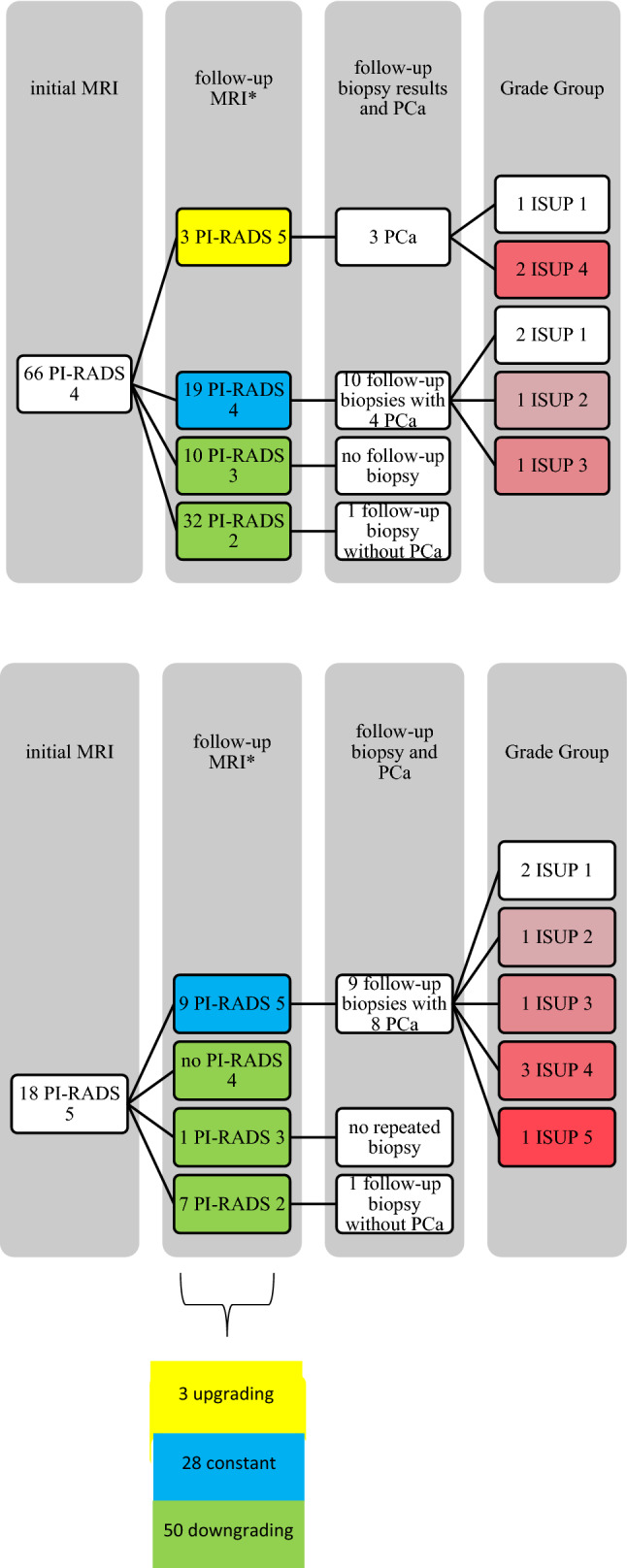
Relation of initial MRI and follow-up MRI, the allocation to a follow-up biopsy and the resulting ISUP Grade. (*on follow-up three PI-RADS Scores unknown).

The initial PI-RADS rating changed from 66/84 (78%) PI-RADS 4 and 18 (22%) PI-RADS 5 lesions to 39 (48%) PI-RADS 2 lesions, 11 (13%) PI-RADS 3 lesions, 19 (23%) PI-RADS 4 lesions and 12 (15%) PI-RADS 5 in the follow-up mpMRI (Fig. 3). Overall, a PI-RADS downgrading occurred in 50 (60%) cases and an upgrading in three (4%) cases. A median of 1 (IQR 1–2) lesion per patient was found in mpMRI. The median lesion size was 12 mm (IQR 9–20) (Table 1).

The indication for a follow-up mpMRI was justified by either a rising PSA value (median rise 3.2 ng/ml (IQR 3.9 (1.6–5.4)), a new suspicion in the DRE or a combination of both. Within the subgroup of men with repeated mpMRIs, there were 19/84 (23%) men with more than one additional mpMRI due to a change in the clinical parameter (rising PSA and/or new suspicion in DRE).

60/84 (71%) men received a new MRI but no additional biopsy because of a downgrading of the lesion or a constant finding either in MRI and/ or because of constant clinical parameters.

In total, 32/177 (18%) men had a follow-up transrectal TB within a median of 14 months (IQR 9–26) from the initial TB at our institution (Fig. 1). The PI-RADS scores were PI-RADS 4 in 11 cases and PI-RADS 5 in 10 cases (Table 2). The overall detection rate was 66% (21/32) and csPCA was detected in 14/32 (44%) men. This represents 12% (21/177) and 8% (14/177) of the whole cohort (Fig. 2). Please see Fig. 3 for a summary of the initial MRI, the follow-up and the related biopsy results.

**Table 2:** PI-RADS Score in relation with ISUP in follow-up TB (n = 21).

PI-RADS	n (%)	ISUP 1	ISUP 2	ISUP 3	ISUP 4	ISUP 5
4	11 (52%)	5 (24%)	1 (5%)	1 (5%)	3 (14%)	1 (5%)
5	10 (48%)	2 (9%)	2 (9%)	1 (5%)	2 (10%)	3 (14%)
	21 (100%)	7 (33%)	3 (14%)	2 (10%)	5 (24%)	4 (19%)

The ISUP Group Grade distribution of the follow-up TB was as follows: 7/21 (33%) cases of ISUP 1, 3 (14%) cases of ISUP 2, 2 (10%) men of ISUP 3, 5 (24%) men of ISUP 4 and 4 (19%) with an ISUP 5. The correlation of the ISUP Grade Group and the PI-RADS ratings are likewise shown in Table 2. The chosen treatment is shown in Supplemental 1. After a radical prostatectomy the biopsy GS was upgraded in 15% and downgraded in 31% of the cases (Supplemental 2).

The underlying reasons for no further biopsies were available in 110/177 (62%) men (Supplemental 3). In 71% (78/110) no further biopsy was warranted due to stable clinical parameters such as PSA and DRE. 12% (13/110) of men had left urological care and 7% (8/110) patients did not agree to a new biopsy despite an indication. In 79% (85/110) of the patients, the decision for no further biopsy was made by the treating outpatient urologist and in 21% (23/110) men the decision against a biopsy was based on their own opinion.

## Discussion

Despite the positive impact of the mpMRI on selected men at risk for csPCa not all men with a PI-RADS 4 or 5 lesion have cancer detected in prostate biopsy and their follow-up remains unclear. Currently, there is little data on the real-life management of men after a negative TB approach.

Kinnaird et al. support that a follow-up TB is necessary if suspicious lesions are seen on the follow-up MRI as 54% of the patients had csPCa with a PI-RADS 5 lesion in their cohort, which is in line with our findings^19^. Hence, mpMRI works as a triage-test in biopsy-naïve men and is further highly reliable in excluding significant disease in men with previous negative biopsy^11,20^. Panebianco et al. underline that as 1827 (75% of 2422 in total) patients with positive mpMRI after an initial negative were diagnosed with PCa and 1103 (46%) had csPCa, respectively^21^. At the same time, a MRI may indicate false-positive findings (e.g. PROMIS: positive predictive value 51%, specificity 41%), so that a biopsy may be unnecessary^11,22^.

TB surpasses the SB in terms of detection rate in ISUP Grade > 2 in the repeat-biopsy setting^2^. In our follow-up biopsy setting the cancer detection rate (CDR) in total was 66% (21/32) whereas in a primary setting the CDR for a targeted biopsy alone and for TB in combination with SB was 63% and 74% in our institution in a similar cohort, respectively^6^.

There is little data or guidance on how to manage these men especially if a PI-RADS ≥ 4 lesion was biopsy-negative. Ullrich et al. recommend a re-biopsy of a PI-RADS 4 lesion in the peripheral zone when there has been a negative biopsy before but suggest a follow-up for negatively biopsied transition zone lesions^23^. The updated German guidelines have added a recommendation that no further imaging or biopsy is indicated after a negative TB if clinical parameters are stable^22^.

Additionally, one needs to strongly consider a reevaluation of the MRI and technical issues of the TB itself may have led to false-negative biopsy results. With a broader use of the mpMRI, the known pitfalls such as reader inter-variability, imaging quality and missed TB may lead to an increase of men with negative TB and the need of standardized patient management^24^. As we found a high rate of downgrading (60% (50/84)), a “reference” radiology might give further guidance. One explanation may be prostatic inflammations which mimic cancer lesions on PI-RADS, which can disappear on further follow-up^23,25,26^.

Concordant to our findings Meng et al. established that 88/497 (18%) of patients with a PIRADS 4 or 5 lesion prior to initial biopsy had no cancer in TB^27^ due to a PI-RADS downgrading in 73% of men on repeat MRI^27^. The prospective study by Meng^27^ over 4 years included 497 men with a negative biopsy out of a cohort of 1595 men (overall 31% of men), whereas in our consecutive cohort only 14% (222/1546) of PI-RADS 4/5 cases presented with a negative biopsy result. This finding may be explained by our single center experience with a high primary detection rate^6^.

Persistent PI-RADS 4/5 lesions are at high risk of missed PCa, but false positive findings still exist as e.g. PCa in the transitional zone is still challenging^23,27,28^. Prostate deformation, patient movement, mismatch in imaging may be other reasons for failure in TB^24^.

With a follow-up of 80% and its prospective documentation this study needs further long-term data and potential data of the PRECSION trial will give further insight into risk assessment of patients with a negative biopsy, either SB or TB^12^. The limitations of our study are its retrospective nature and the lack of histological proof of absence of cancer in all men. A further limitation is, that the analysis presents the real-world follow up data and clinical decision were not based on a standard follow-up protocol and may therefore underestimate the absence on csPCa during follow-up.

## Conclusion

Our study presents real world data on the management of men with a negative TB biopsy. Men with a positive mpMRI and lesions with high suspicion (PI-RADS4/5) and a negative targeted biopsy should be critically reviewed and considered for repeat biopsy or strict surveillance. The optimal clinical risk assessment remains to be further evaluated.

## Supplementary Information

Supplementary Information.

## Data Availability

The datasets used and/or analysed during the current study available from the corresponding author on reasonable request.
