# Supplementary material for: Follow-up of men with a PI-RADS 4/5 lesion after negative MRI/Ultrasound fusion biopsy
Source: Sci Rep. 2022 Aug 10;12:13603. doi: 10.1038/s41598-022-17260-6 (PMC9365776; doi:10.1038/s41598-022-17260-6)
Supplement: Supplementary file 1 — Supplementary Information. [file 41598_2022_17260_MOESM1_ESM.docx]

**Supplementals:**

**Supplemental 1:** Chosen treatment after follow-up biopsy

| Number of patients (n) | Chosen treatment |
| --- | --- |
| 9 | **Robotic assisted radical prostatectomy (RARP); please see Supplemental 2** |
| 9 | **Active Surveillance** |
| 2 | **External beam radiation therapy (EBRT)** |
| 1 | **High-intensity focused ultrasound (HIFU)** |

**Supplemental 2:** Histopathological Stage after radical prostatectomy (n=39)

| Histopathological stage | n (%) |
| --- | --- |
| pT2b | **1 (8)** |
| pT2c | **7 (54)** |
| pT3a | **4 (30)** |
| pT3b | **1 (8)** |
| pN0 | **12 (92)** |
| pN1 | **1 (8)** |
| R0 | **8 (62)** |
| R1 | **5 (38)** |

**Supplemental 3:** Reasons for no follow-up biopsy (n=110)

|  | n | % |
| --- | --- | --- |
| Not necessary (includes stable PSA and no changing in the examination like DRE) | 78 | 71 |
| Patient doesn’t want to | 8 | 7 |
| Patient lost to follow-up (not in urological care anymore) | 13 | 12 |
| Patient prefers MRI (already planned) | 2 | 2 |
| New biopsy scheduled | 4 | 3 |
| Patient died before biopsy | 4 | 3 |
| Other | 1 | 1 |
